# Special Issue: Recent Advances in Microglia Research

**DOI:** 10.3390/ijms26020507

**Published:** 2025-01-09

**Authors:** Daniele Lana, Maria Grazia Giovannini

**Affiliations:** Department of Health Sciences, Section of Clinical Pharmacology and Oncology, University of Florence, Viale Pieraccini 6, 50139 Firenze, Italy; mariagrazia.giovannini@unifi.it

This Editorial introduces the new Special Issue, published in the *International Journal of Molecular Sciences* and entitled “Recent Advances in Microglia Research”, which covers this important topic with a collection of five valuable contributions: three Original Research Articles and two Reviews.

Microglia, the primary immune cells of the central nervous system, have very mobile ramified branches that dynamically and continuously survey brain parenchyma to detect and eliminate debris of damaged neurons via phagocytosis and to restore tissue homeostasis. Microglia are plastic cells that undergo profound functional reprogramming and actively maintain their protective role during normal aging, but this ability is considerably decreased in a pro-inflammatory context.

An oversimplified view recognizes two extremes of profound functional reprogramming in response to cytokines, chemokines, and other soluble factors produced by damaged neurons, the classical pro-inflammatory and the anti-inflammatory phenotypes, but a plethora of intermediate phenomics have been found between these extreme functional states, as described by Paolicelli et al. [1]. The heterogeneity of microglia, either between or within a particular brain region, is likely relevant in healthy conditions and disease processes [2,3]. Understanding the spatial and temporal differences and roles of microglia will help to determine how the interactions between microglia, astrocytes, and neurons might influence the state and progression of diseases, and will be paramount in identifying therapeutic strategies. The complexity of microglia phenotypes and their related functions necessitates the continuous study of microglia in health and disease [4]. The investigators that have contributed to this Special Issue with original research articles and reviews are improving the understanding of the role of microglia in various physiological conditions, in normal brain aging, and in neurodegenerative diseases.

Palahati et al. [5] investigated whether microglia transcription factors contribute to the inflammatory response after intracerebral hemorrhage (ICH) [6,7]. Using bioinformatic screening, they identified the potential transcription factor tonicity-responsive enhancer-binding protein (TonEBP) in microglia. Recently, the group discovered that ICH causes an increase in TonEBP in microglia in peri-hematoma regions of both human and mouse brains, and the KO of TonEBP mitigates LPS-induced inflammation and NF-κB signaling activation in microglia. The authors sequenced the transcriptomes of TonEBP-deficient cells and showed that TonEBP may act as a transcription factor of PELI1, which has been reported to be a regulator of the NF-κB pathway [8]. Moreover, they demonstrated that the knockdown of microglial TonEBP could regulate the NF-κB pathway and attenuate the inflammation response. Therefore, they concluded that TonEBP may mediate the regulation of the NF-κB pathway and the inflammatory response, promoting the expression of PELI1. This research elucidates part of the underlying mechanism of TonEBP in inflammation and can provide a potential therapeutic target against the ICH inflammatory response. The authors concluded that the role of microglial TonEBP in neuroinflammation after a hemorrhagic stroke in mice deserves further attention.

Virtuoso et al. [9] mechanically induced a lesion of the Schaffer collaterals (SCL) in mouse entorhino–hippocampal slice cultures to investigate microglia and astrocytes behavior in CA3, the lesioned area, and in CA1, the denervated area, and studied metalloproteinases (MMPs) modulation [10]. Three days after the lesion, they observed distinct response patterns in microglia and astrocytes. Interestingly, while GFAP-expressing astrocytes showed no immediate changes after SCL, microglia responses varied depending on their anatomical location, underscoring the complexity of the post-injury hippocampal neuroglial network. The MMPs inhibitor GM6001 increased the number of Iba1-expressing cells and caused a withdrawal of their primary branches in CA1, but did not affect microglia reactions in CA3. The present study shows that glial responses differ on the basis of the specific CNS region involved, the nature of the initiating event, and the timing after the insult [11,12]. Understanding the region-specific and time-dependent dynamics of glial cells in pathological conditions will provide deeper insights into both adaptive and maladaptive processes. These findings highlight the importance of understanding glia regionalization following neural injury and MMPs modulation and pave the way for further research into glia-targeted therapeutic strategies for neurodegenerative disorders [13].

Zohar et al. [14] used a primary neonatal microglia culture as an in vitro model to test drugs that may interact with inflammatory signaling and the lncRNA regulatory network [15,16]. In this work, they stimulated murine neonatal microglia cells with benzoyl ATP (bzATP) and LPS and monitored their ability to release pro-inflammatory cytokines. Cells exposed to bzATP, a purinergic receptor agonist, undergo a short-lived wave of transcriptional changes. However, a sustainable and robust response was found only when bzATP was administered together with LPS. Several abundant long noncoding RNAs (lncRNAs) that function in inflammation and cytokine production, such as Ptgs2os2, Bc1, and Morrbid, are induced by bzATP/LPS. Analyzing the observed changes through the TNF and NF-kB pathways, the authors confirmed that neonatal glial cells exhibit a distinctive expression program in which inflammatory-related genes are highly upregulated. The observed capacity of the microglia culture to activate a robust inflammatory response is useful for studying neurons under stress, following brain injury, and during aging [17]. The authors propose the use of a primary neonatal microglia culture as a responsive in vitro model for testing drugs that may interact with inflammatory signaling and the lncRNA regulatory network.

The review by Chagas and Serfaty [18] discusses the involvement of microglia in the neuroinflammatory mechanisms that represent known key elements in the pathogenesis of COVID-19. Indeed, the dysregulation of microglia function can severely impact both functional and structural plasticity, leading to the cognitive sequelae that appear in the pathogenesis of Long COVID [19,20]. Microglia, the immune cells in the central nervous system and key elements regulating brain development and brain health, are fully responsive to stressors and to microenvironmental alterations [21,22]. Furthermore, they are involved in the construction of neural circuits and full experience-dependent plasticity. Therefore, understanding this complex scenario is mandatory when establishing the possible molecular mechanisms correlated with Long COVID symptoms. The authors discuss Long COVID and its association with reduced levels of BDNF, altered crosstalk between circulating immune cells and microglia, increased levels of inflammasomes, cytokines and chemokines, and alterations in the signaling pathways that impact neural synaptic remodeling and plasticity, such as fractalkines, the complement system, the expression of SIRPα and CD47 molecules, and altered matrix remodeling. These complex mechanisms may help us to understand the consequences of Long COVID for brain development and plasticity, and how it may cause neurodevelopmental disorders, learning disabilities, and cognitive decline in adults [23].

Lana et al. [24] discuss the literature on the alterations in microglia phenomics in the hippocampus of animal models of normal brain aging, acute neuroinflammation, ischemia, and AD. Microglia undergo phenomic modifications consisting of transcriptional, functional, and morphological changes that transform them into cells with different properties and functions. They describe the phenomic modifications of microglia, focusing on the diverse models of neurodegenerative disorders, as well as modifications that occur in different areas of the brain. For instance, in contiguous and highly interconnected regions of rat hippocampus, microglia show a differential, finely regulated, and region-specific reactivity, demonstrating that their responses to insults are not uniform but vary significantly from area to area [25,26]. It is of great interest to verify whether the differences in microglia reactivity explain the regional heterogeneous responses to inflammatory stimuli [27]. Understanding the spatiotemporal differences in microglia phenomics in health and disease is of paramount importance to find new targets for the development of novel microglia-oriented therapies in different CNS disorders. The interventions have at least two different aims: (i) suppression of the pro-inflammatory properties of microglia to limit the deleterious effect of their activation; (ii) modulation of phenotypic microglia changes to improve their anti-inflammatory properties.

These contributions further highlight the importance of continuing research on the different aspects of microglia responses in the brain, which possibly represent a basis for novel and viable strategies for the prevention and treatment of neurodegenerative diseases.
